# Supplementary material for: Size-dependent loss of aboveground animals differentially affects grassland ecosystem coupling and functions
Source: Nat Commun. 2018 Sep 11;9:3684. doi: 10.1038/s41467-018-06105-4 (PMC6133970; doi:10.1038/s41467-018-06105-4)
Supplement: Supplementary file 1 — Supplementary Information [file 41467_2018_6105_MOESM1_ESM.pdf]

Supplementary Figures

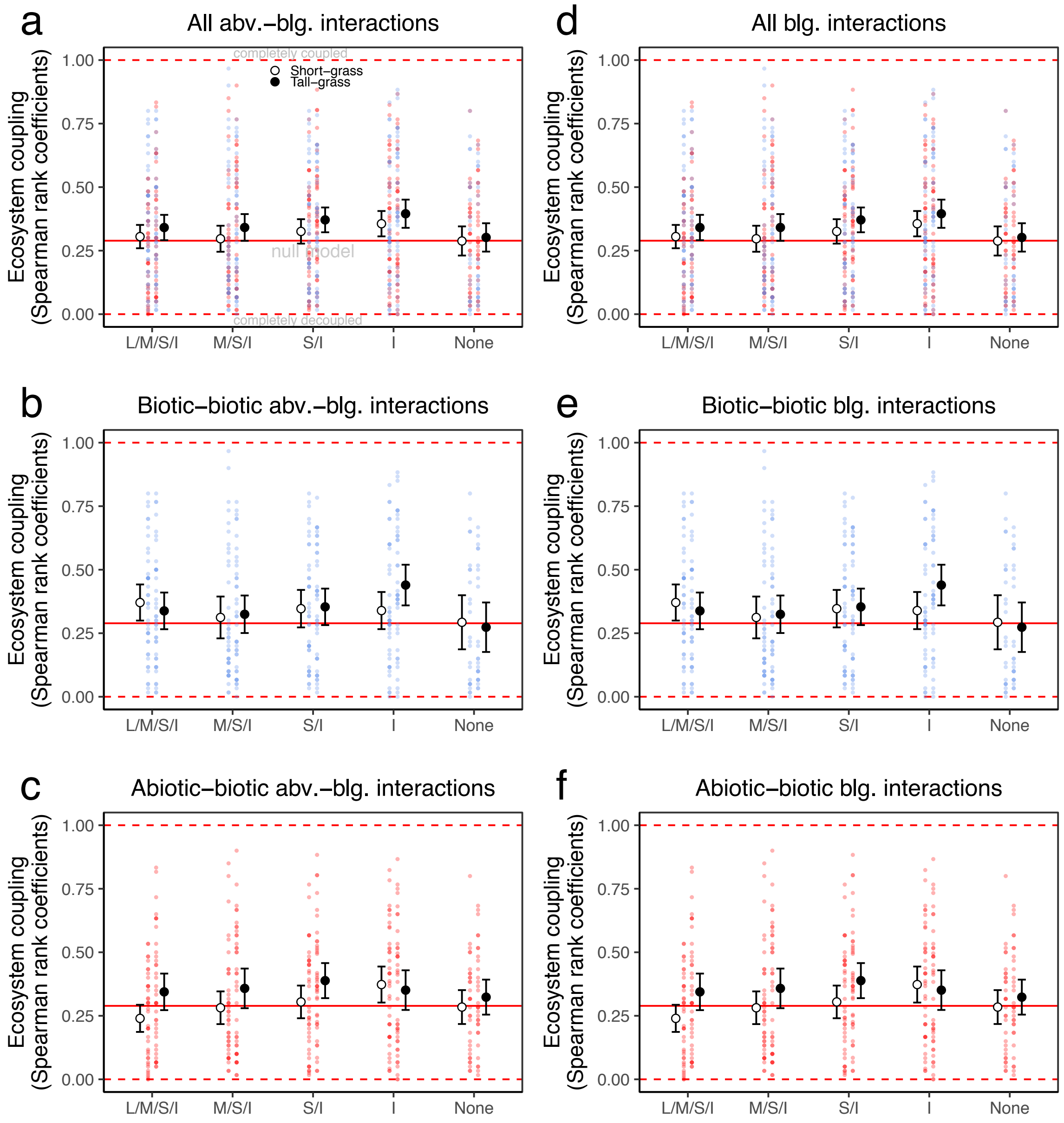

Supplementary Fig 1. Effect of progressive animal exclusion with size-selective fences on ecosystem coupling by vegetation type. Ecosystem coupling (absolute values of Spearman's rho) calculated by vegetation type based on (a) all interactions, (b) biotic-biotic interactions, (c) abiotic-biotic interactions involving above- and belowground constituents, and (d) all interactions, (e) biotic-biotic interactions, (f) abiotic-biotic soil interactions involving belowground constituents only. All above-belowground interactions: n = 80 (n = 56 in the case of the "None" treatment). Biotic-biotic above-belowground interactions: n = 40 (n = 24 in the case of the "None" treatment). Abiotic-biotic above-belowground interactions: n = 40 (n = 32 in the case of the "None" treatment). All belowground interactions: n = 36. Biotic-biotic belowground interactions: n = 12. Abiotic-biotic belowground interactions: n = 24. Red line: null model below which average correlation happens by chance. Red dashed lines: greatest/smallest coupling values possible. Error bars: 95% confidence interval of the mean. Background points: individual interactions between biotic-biotic (blue) and abiotic-biotic (red) constituents. "L/M/S/I": Large/medium/small mammals, and invertebrates have access, "M/S/I": Medium/small mammals, and invertebrates have access, "S/I": Small mammals and invertebrates have access, "I": Invertebrates have access, "None": No animals have access (see Fig 1). Short-grass (open symbols) = short-grass vegetation, Tall-grass (closed symbols) = tall-grass vegetation. Abv. = aboveground, blg = belowground.

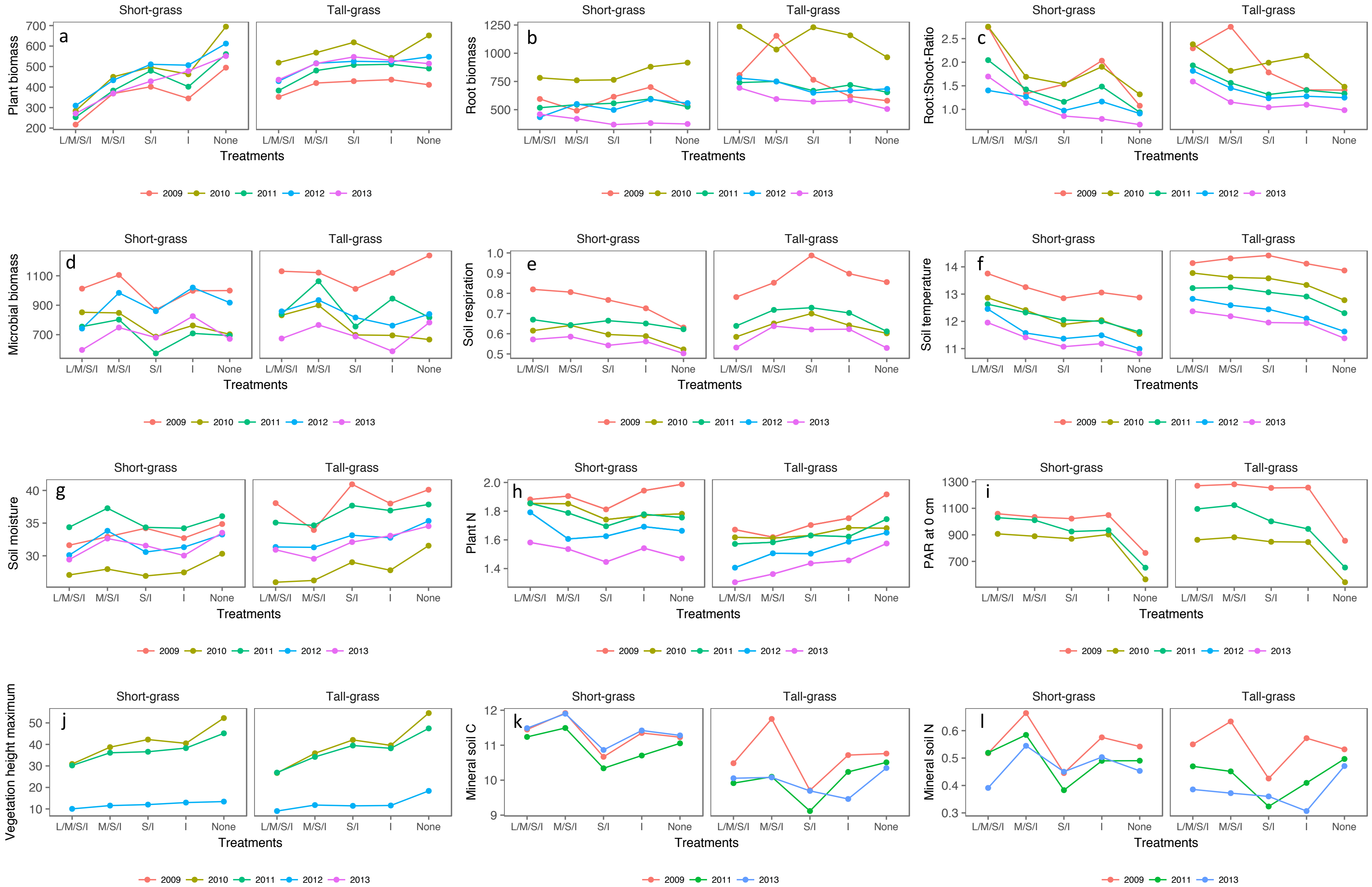
Short-grass
Tall-grass
a
b
c
d
e
f
g
h
i
j
k
l
Plant biomass
Root biomass
Root:Shoot-Ratio
Microbial biomass
Soil respiration
Soil temperature
Soil moisture
Plant N
PAR at 0 cm
Vegetation height maximum
Mineral soil C
Mineral soil N
L/M/S/I
M/S/I
S/I
I
None
Treatments
2009
2010
2011
2012
2013

Supplementary Figure 2. Temporal development of selected environmental, plant and soil variables measured in our experimental treatments. Variables that responded to our treatment already did so after the first growing season. (a) Plant biomass = Aboveground plant biomass (g m^{-2}), (b) Root biomass (g m^{-2}), (c) Root:shoot ratio = ratio of root biomass to aboveground plant biomass, (d) Microbial biomass carbon = microbial biomass C (mg kg^{-1} soil), (e) Soil respiration (g m^{-2} h^{-1}), (f) Soil temperature (°C), (g) Soil moisture (%), (h) Plant N = Plant tissue N content (%), (i) PAR at 0 cm = photosynthetically active radiation (PAR) at the soil surface (µmol m^{-2} s^{-1}), (j) Vegetation height maximum = Maximum vegetation height (cm), (k) Mineral soil C = mineral soil C content (%), (l) Mineral soil N = mineral soil N content (%). Different colours are for different years of the experiment (2009 through 2013). Note that not all variables were measured every year. "L/M/S/I": Large/medium/small mammals, and invertebrates have access, "M/S/I": Medium/small mammals, and invertebrates have access, "S/I": Small mammals and invertebrates have access, "I": Invertebrates have access, "None": No animals have access (see also Fig 1). Short-grass = short-grass vegetation, Tall-grass = tall-grass vegetation.

Supplementary Tables

Supplementary Table 1. List of mammalian and invertebrate species that our size-selective fencing treatments excluded. No attempt was made to count large to small mammals within the treatment plots. Invertebrate taxa were identified to the lowest taxonomic level feasible based on morphology and represent all taxa collected in our treatment plots using pitfall traps and suction sampling. Our exclosure design was aimed at excluding mammalian herbivores, but naturally also excluded the few medium and small mammalian predators, as well as the entire aboveground invertebrate food web. Large apex predators were absent or transient (wolf, bear, lynx) from the SNP and reptiles, amphibians and birds are scarce to absent in the subalpine grasslands under study.

**Taxonomic group**	**Feeding type**
Large mammals	
Artidodactyla: *Cervus elaphus*	Herbivore
Artidodactyla: *Rupicapra rupicapra*	Herbivore
Artidodactyla: *Capreolus capreolus*	Herbivore
Artidodactyla: *Capra ibex*	Herbivore
Medium mammals:	
Lagomorpha: *Lepus timidus*	Herbivore
Rodentia: *Marmota marmota*	Herbivore
Carnivora: *Martes foina*	Predator
Carnivora: *Martes martes*	Predator
Carnivora: *Meles meles*	Omnivore
Carnivora: *Mustela erminea*	Predator
Carnivora: *Mustela putorius*	Predator
Carnivora: *Vulpes vulpes*	Omnivore
Small mammals:	
Rodentia: *Apodemus alpicola*	Herbivore
Rodenita: *Apodemus flavicollis*	Herbivore
Rodentia: *Apodemus sylvaticus*	Herbivore
Rodentia: *Chionomys nivalis*	Herbivore
Rodentia: *Clethrionomys glarolus*	Herbivore
Rodentia: *Dryomys nitedula*	Herbivore
Rodentia: *Eliomys quercinus*	Herbivore
Rodentia: *Microtus agrestis*	Herbivore
Rodentia: *Microtus arvalis*	Herbivore
Rodentia: *Rattus norvegicus*	Omnivore
Carnivora: *Mustela nivalis*	Predator
Soricomorpha: *Neomys fodiens*	Predator
Soricomorpha: *Sorex araneus*	Predator
Soricomorpha: *Sorex minutus*	Predator
Invertebrates	
Araneae	Predator
Blattodea	Omnivore
Chilopoda	Predator
Coleoptera	Mixed group
Coleoptera: Brentidae	Herbivore
Coleoptera: Buprestidae	Herbivore
Coleoptera: Byrrhidae	Herbivore
Coleoptera: Cantharidae	Predator
Coleoptera: Carabidae	Predator
Coleoptera: Cholevinae	Detritivore

Supplementary Table 1 (continued).

Taxonomic group	Feeding type
Coleoptera: Chrysomelidae	Herbivore
Coleoptera: Coccinellidae	Mixed group
Coleoptera: Coloninae	Detritivore
Coleoptera: Colydiinae	Herbivore
Coleoptera: Cryptophagidae	Detritivore
Coleoptera: Curculionidae	Herbivore
Coleoptera: Dascillidae	Unknown
Coleoptera: Dasytidae	Mixed group
Coleoptera: Elateridae	Omnivore
Coleoptera: Eucnemidae	Unknown
Coleoptera: Geotrupidae	Detritivore
Coleoptera: Histeridae	Predator
Coleoptera: Hydraenidae	Herbivore
Coleoptera: Hydrophilidae	Detritivore
Coleoptera: Lampyridae	Adults do not eat
Coleoptera: Latridiidae	Detritivore
Coleoptera: Leiodidae	Detritivore
Coleoptera: Nitidulidae	Mixed group
Coleoptera: Pselaphinae	Predator
Coleoptera: Ptiliidae	Detritivore
Coleoptera: Ptininae	Detritivore
Coleoptera: Scarabaeoidea	Mixed group
Coleoptera: Scolytinae	Herbivore
Coleoptera: Scydmaenidae	Predator
Coleoptera: Staphylinidae	Predator
Coleoptera: Tenebrionidae	Detritivore
Dermaptera	Omnivore
Diplopoda	Detritivore
Diptera	Mixed group
Hemiptera: Aphidoidea	Herbivore
Hemiptera: Auchenorrhyncha	Herbivore
Hemiptera: Coccoidea	Herbivore
Hemiptera: Heteroptera	Mixed group
Hemiptera: Psyllidae	Herbivore
Hymenoptera	Mixed group
Hymenoptera: Formicidae	Omnivore
Isopoda	Detritivore
Lepidoptera	Nectar feeder
Mollusca	Herbivore
Oligochaeta	Detritivore
Opiliones	Omnivore
Orthoptera	Herbivore
Orthoptera: Acrididae	Herbivore
Orthoptera: Gryllidae	Omnivore
Orthoptera: Tetrigidae	Herbivore
Orthoptera: Tettigoniidae	Herbivore
Pseudoscorpiones	Predator
Psocoptera	Detritivore
Thysanoptera	Herbivore

11

Supplementary Table 2. Mean (standard error) values of our four abiotic ecosystem constituents used to calculate ecosystem coupling as well as six individual ecosystem functions to calculate ecosystem multifunctionality. Units: fine fraction bulk density = g cm^{-3}, soil moisture = %, soil organic C content = %, soil net N mineralisation = kg ha^{-1} $season^{-1}$, soil respiration = g m^{-2} h^{-1}, plant tissue N content = %, root biomass = g m^{-2}, microbial biomass C = mg kg^{-1} soil. “L/M/S/I”: Large/medium/small mammals, and invertebrates have access, “M/S/I”: Medium/small mammals, and invertebrates have access, “S/I”: Small mammals and invertebrates have access, “I”: Invertebrates have access, “None”: No animals have access (see also Fig 1).

	Short-grass vegetation					Tall-grass vegetation				
	L/M/S/I	M/S/I	S/I	I	None	L/M/S/I	M/S/I	S/I	I	None
Fine fraction bulk density	0.6 (0.1)	0.6 (0.1)	0.7 (0.1)	0.7 (0.2)	0.8 (0.1)	0.6 (0.1)	0.7 (0.1)	0.5 (0.1)	0.6 (0.1)	0.5 (0.1)
Soil moisture	27.5 (2. 5)	26.3 (1.9)	28.5 (2.3)	29.4 (2.8)	30.7 (2.2)	25.7 (2.5)	28.6 (2.7)	27.6 (2.4)	26.3 (2.8)	29.3 (2.3)
Soil pH	7.05 (0.1)	7.1 (0.1)	7.1 (0.1)	7.1 (0.1)	7.2 (0.1)	7.1 (0.1)	6.8 (0.2)	7.1 (0.1)	7.0 (1.0)	7.0 (0.1)
Soil organic C content	17.3 (1.7)	18.5 (2.3)	17.1 (1.9)	18.3 (2.5)	17.5 (2.1)	15.8 (1.8)	16.2 (1.9)	17.9 (2.3)	16.2 (1.9)	17.5 (2.5)
Soil net N mineralisation	0.5 (0.1)	0.9 (0.1)	0.9 (0.1)	0.9 (0.1)	0.8 (0.1)	0.8 (0.1)	0.9 (0.2)	0.9 (0.1)	1.1 (0.2)	0.9 (0.1)
Soil respiration	0.5 (0.1)	0.6 (0.04)	0.6 (0.04)	0.6 (0.1)	0.5 (0.04)	0.6 (0.04)	0.6 (0.1)	0.5 (0.03)	0.6 (0.03)	0.5 (0.04)
Plant tissue N content	1.6 (0.04)	1.5 (0.04)	1.5 (0.1)	1.5 (0.1)	1.5 (0.02)	1.3 (0.1)	1.4 (0.04)	1.4 (0.02)	1.5 (0.04)	1.6 (0.04)
Plant species richness	35 (1.3)	30.6 (2.5)	31.1 (1.8)	29.7 (2.4)	28.8 (2.8)	36.6 (1.3)	36 (1.2)	35.6 (2.0)	33.8 (2.0)	34.8 (2.1)
Root biomass	460 (85)	419 (31.3)	368 (48)	381 (68)	373 (47)	693 (88)	595 (26)	571 (69)	583 (50)	506 (37)
Microbial biomass C	596 (73)	748 (116)	680 (48)	826 (131)	671 (135)	674 (104)	766 (145)	688 (86)	587 (81)	782 (138)

Supplementary Table 3. Permutation based (n = 999) p-values indicating whether ecosystem coupling was significantly greater than the null model. Values are provided for both vegetation types together (All) and short- (Short-grass) and tall-grass (Tall-grass) vegetation separately for all interactions, biotic-biotic interactions, abiotic-biotic interactions involving above- and belowground constituents, and all interactions, biotic-biotic interactions, abiotic-biotic soil interactions involving belowground constituents only. Values in bold and red represent p-values < 0.05, values in italic and blue represent p-values between 0.05 and 0.1. "L/M/S/I": Large/medium/small mammals, and invertebrates have access, "M/S/I": Medium/small mammals, and invertebrates have access, "S/I": Small mammals and invertebrates have access, "I": Invertebrates have access, "None": No animals have access (see also Fig 1). Short-grass = short-grass vegetation, Tall-grass = tall-grass, Veg type = Vegetation type.

p-values

**Treatment**	**Veg type**	**Above-belowground**			**Belowground**		
		All	*Biotic-biotic*	*Abiotic-biotic*	*All*	*Biotic-biotic*	*Abiotic-biotic*
L/M/S/I	All	**0.014**	**0.002**	0.436	0.287	*0.062*	0.692
M/S/I	All	**0.039**	**0.041**	**0.036**	0.207	0.632	*0.098*
S/I	All	**<0.001**	**<0.001**	**0.001**	**0.046**	0.671	**0.004**
I	All	**<0.001**	**<0.001**	**0.001**	**0.006**	**<0.001**	*0.063*
None	All	0.338	0.338	0.338	0.219	0.219	0.219
L/M/S/I	Short-grass	*0.065*	**0.006**	0.416	0.343	**0.029**	0.639
M/S/I	Short-grass	0.330	0.147	0.590	0.440	0.906	0.193
S/I	Short-grass	*0.059*	**0.012**	0.232	0.162	0.427	*0.088*
I	Short-grass	**0.005**	**0.023**	**0.001**	**0.032**	0.642	**0.006**
None	Short-grass	0.760	0.681	0.808	0.728	0.513	0.903
L/M/S/I	Tall-grass	*0.072*	**0.004**	0.421	0.356	**0.036**	0.686
M/S/I	Tall-grass	**0.019**	*0.069*	**0.005**	0.158	0.211	0.135
S/I	Tall-grass	**<0.001**	**0.005**	**<0.001**	*0.066*	0.799	**0.008**
I	Tall-grass	**<0.001**	**<0.001**	**0.005**	**0.030**	**<0.001**	0.780
None	Tall-grass	0.609	0.923	0.514	0.536	0.705	0.508

Supplementary Table 4. Correlations between ecosystem coupling and individual ecosystem functions. Pearson correlations are presented for all interactions, biotic-biotic interactions, abiotic-biotic interactions involving all above- and belowground constituents, and all interactions, biotic-biotic interactions, abiotic-biotic soil interactions involving belowground constituents only. N = 10. Numbers in bold denote statistically significant correlations. Units for functions: soil net N mineralisation = kg ha^{-1} $season^{-1}$, soil respiration = g m^{-2} h^{-1}, plant tissue N content = %, root biomass = g m^{-2}, microbial biomass C = mg kg^{-1} soil.

	**Ecosystem coupling**											
	Above-belowground						Belowground					
**Individual ecosystem functions**	*All interactions*		*Biotic-biotic interactions*		*Abiotic-biotic interactions*		*All interactions*		*Biotic-biotic interactions*		*Abiotic-biotic interactions*	
	rho	P-value	rho	P-value	rho	P-value	rho	P-value	rho	P-value	rho	P-value
Soil net N mineralisation	**0.67**	**0.03**	0.31	0.38	**0.70**	**0.02**	**0.82**	**0.004**	0.35	0.31	0.33	0.35
Soil respiration	0.13	0.71	0.11	0.75	0.11	0.75	0.14	0.69	-0.27	0.46	0.34	0.33
Plant tissue N content	-0.39	0.26	-0.14	0.69	-0.47	0.17	-0.25	0.48	-0.19	0.60	-0.03	0.93
Plant species richness	0.34	0.34	0.22	0.54	0.28	0.44	0.17	0.64	0.22	0.55	-0.06	0.88
Root biomass	0.51	0.13	0.28	0.43	0.48	0.16	0.34	0.34	0.39	0.27	-0.07	0.85
Microbial biomass C	-0.17	0.64	**-0.66**	**0.03**	0.37	0.29	0.22	0.55	**-0.68**	**0.03**	**0.76**	**0.01**

Supplementary Table 5: Relationships at the vegetation type by treatment level (n=10) between pairwise correlation coefficients of biotic and abiotic constituents and individual ecosystem functions/multifunctionality. Pairwise interactions between constituents are based on nine replicates each. Values represent Spearman rank correlation coefficient. Values in bold and red represent significant correlations with p-values < 0.05, values in italic and blue represent significant correlations with p-values between 0.05 and 0.1. Soil C = soil organic C content, Nematodes = soil nematode community, Microbes = soil microbial community, Soil arthro = soil arthropod community, Abv inv = Aboveground invertebrate community, Plants = Vascular plant community, PC1 = Community composition of a respective community based on PCA axis 1, PC2 = Community composition of a respective community based on PCA axis 2. Net N min = mineral soil net N mineralisation, Soil resp = soil respiration, Plant N = plant tissue N content, Abv = aboveground, Rich = plant species richness, MBC = microbial biomass carbon, Multi-func. = Multifunctionality

	Net N min	Soil resp	Plant N	Rich	Root biomass	MBC	Multi-func
Nematodes PC1 - BD	0.148	0.841	0.126	0.185	**0.021**	0.700	**0.012**
Nematodes PC2 - BD	0.555	0.166	0.383	0.185	*0.080*	0.532	0.154
Mircobes PC1 - BD	0.947	0.663	0.327	**0.032**	0.250	0.354	0.393
Mircobes PC2 - BD	0.960	**0.001**	0.247	0.530	0.762	0.410	0.541
Abv inv PC1 - BD	0.588	**0.011**	0.844	0.168	0.146	*0.069*	0.399
Abv inv PC2 - BD	0.610	0.233	**0.047**	**0.021**	**0.015**	0.823	**0.047**
Soil arthro PC1 - BD	0.814	0.853	0.108	0.400	0.113	*0.099*	0.224
Soil arthro PC2 - BD	0.533	**0.013**	0.751	0.556	0.162	0.328	0.293
Plants PC1 - BD	0.533	0.467	0.556	0.276	*0.082*	0.150	0.200
Plants PC2 - BD	0.866	0.160	0.960	0.252	0.148	0.735	0.428
Nematodes PC1 - SWC	0.106	0.688	**0.037**	0.199	0.199	0.947	**0.047**
Nematodes PC2 - SWC	0.223	0.906	0.379	0.461	0.440	*0.093*	0.987
Mircobes PC1 - SWC	0.244	0.651	*0.074*	*0.060*	**0.048**	0.385	**0.022**
Mircobes PC2 - SWC	0.829	0.960	*0.074*	0.907	0.651	0.855	0.651
Abv inv PC1 - SWC	*0.069*	0.933	0.866	0.509	0.756	0.821	0.453
Abv inv PC2 - SWC	0.493	0.233	0.320	**0.047**	**0.002**	0.456	**0.037**
Soil arthro PC1 - SWC	0.651	0.726	0.347	0.385	0.489	*0.067*	0.556
Soil arthro PC2 - SWC	0.108	0.260	0.174	0.260	*0.067*	0.987	**0.029**
Plants PC1 - SWC	0.676	0.580	0.676	0.150	**0.022**	0.751	*0.082*
Plants PC2 - SWC	0.575	0.827	0.623	0.379	0.697	**0.014**	0.551
Mircobes PC1 - Nematodes PC1	0.403	0.455	0.143	**0.044**	**0.024**	0.345	**0.026**
Mircobes PC2 - Nematodes PC1	0.489	0.489	0.405	0.603	0.855	0.365	0.627
Abv inv PC1 - Nematodes PC1	0.799	0.548	0.317	0.866	0.608	0.108	0.955
Abv inv PC2 - Nematodes PC1	0.207	0.233	0.911	0.493	0.207	0.493	0.183
Soil arthro PC1 - Nematodes PC1	0.126	0.713	0.789	0.881	0.336	0.934	0.154
Soil arthro PC2 - Nematodes PC1	0.402	0.577	0.737	**0.030**	**0.012**	0.343	*0.052*
Plants PC1 - Nematodes PC1	*0.088*	0.586	0.550	0.893	0.329	0.329	0.179
Plants PC2 - Nematodes PC1	0.829	*0.067*	0.627	**0.029**	*0.060*	0.580	0.260
Soil C - Nematodes PC1	0.934	0.651	**0.009**	0.200	0.162	0.128	0.200
pH - Nematodes PC1	0.467	0.385	0.347	0.651	0.627	0.907	0.751
Mircobes PC1 - Nematodes PC2	*0.054*	0.108	0.467	0.467	0.138	0.907	*0.060*
Mircobes PC2 - Nematodes PC2	0.347	0.405	0.803	0.174	0.701	0.365	0.960
Abv inv PC1 - Nematodes PC2	**0.021**	0.570	0.693	0.610	0.160	0.911	**0.015**
Abv inv PC2 - Nematodes PC2	**0.037**	0.651	0.736	0.867	0.779	0.610	0.183
Soil arthro PC1 - Nematodes PC2	*0.056*	0.763	0.345	0.841	0.413	0.154	0.199
Soil arthro PC2 - Nematodes PC2	0.894	0.154	0.345	0.213	0.185	0.206	0.126

Supplementary Table 5 (continued)

Plants PC1 - Nematodes PC2	0.802	0.243	0.947	0.815	0.283	1.000	0.555
Plants PC2 - Nematodes PC2	0.663	*0.077*	0.802	0.327	0.763	0.434	0.789
Soil C - Nematodes PC2	0.187	0.627	0.533	0.855	0.907	0.276	0.603
pH - Nematodes PC2	0.275	0.393	**0.032**	0.121	0.258	0.802	*0.066*
Abv inv PC1 - Mircobes PC1	0.670	0.509	*0.091*	0.453	0.435	0.217	0.204
Abv inv PC2 - Mircobes PC1	0.320	0.493	0.385	0.420	0.823	0.651	0.493
Soil arthro PC1 - Mircobes PC1	*0.072*	0.802	0.815	0.402	0.649	0.789	0.531
Soil arthro PC2 - Mircobes PC1	0.803	0.627	0.676	0.467	0.676	0.162	0.960
Plants PC1 - Mircobes PC1	0.434	0.148	0.638	0.510	0.614	0.143	0.476
Plants PC2 - Mircobes PC1	**0.049**	0.613	0.325	1.000	0.880	0.907	0.422
Soil C - Mircobes PC1	0.675	0.434	0.675	0.111	**0.042**	0.258	0.345
pH - Mircobes PC1	0.580	0.829	0.467	0.310	0.187	0.580	0.138
Abv inv PC1 - Mircobes PC2	0.955	*0.069*	0.230	**0.020**	**0.011**	*0.076*	0.192
Abv inv PC2 - Mircobes PC2	**0.037**	0.610	0.955	0.911	0.736	0.651	0.233
Soil arthro PC1 - Mircobes PC2	0.973	0.673	0.960	0.176	0.271	0.163	0.507
Soil arthro PC2 - Mircobes PC2	0.544	0.688	1.000	0.555	0.213	0.275	0.148
Plants PC1 - Mircobes PC2	0.894	0.250	0.137	0.614	0.413	**0.039**	0.675
Plants PC2 - Mircobes PC2	0.533	0.511	0.260	**0.001**	**0.038**	0.511	*0.054*
Soil C - Mircobes PC2	0.440	*0.056*	0.270	0.340	0.359	0.270	0.208
pH - Mircobes PC2	0.934	0.701	0.138	0.751	0.934	0.676	0.987
Soil arthro PC1 - Abv inv PC1	0.548	0.146	0.608	0.417	0.204	0.192	0.286
Soil arthro PC2 - Abv inv PC1	0.978	**0.023**	0.910	0.157	0.365	0.257	0.821
Plants PC1 - Abv inv PC1	0.160	0.207	0.823	0.456	0.867	0.693	0.420
Plants PC2 - Abv inv PC1	0.955	0.260	**0.047**	**0.021**	**0.007**	0.352	0.102
Soil C - Abv inv PC1	0.260	0.207	0.233	*0.058*	**0.007**	0.651	**0.015**
pH - Abv inv PC1	0.493	0.651	**0.037**	**0.047**	**0.004**	0.823	**0.007**
Soil arthro PC1 - Abv inv PC2	0.865	*0.066*	0.798	0.213	0.346	0.606	0.668
Soil arthro PC2 - Abv inv PC2	0.693	0.823	**0.015**	0.120	0.160	0.779	0.139
Plants PC1 - Abv inv PC2	0.136	0.490	0.317	0.146	0.382	0.933	0.888
Plants PC2 - Abv inv PC2	0.493	0.385	**0.007**	**0.028**	*0.071*	0.823	*0.071*
Soil C - Abv inv PC2	**0.040**	0.844	*0.091*	0.243	**0.050**	0.778	**>0.001**
pH - Abv inv PC2	**0.001**	0.610	0.736	0.289	0.651	0.610	0.320
Plants PC1 - Soil arthro PC1	*0.084*	0.854	0.713	0.907	0.894	0.111	0.476
Plants PC2 - Soil arthro PC1	0.200	0.385	0.174	0.244	0.174	0.651	*0.054*
Soil C - Soil arthro PC1	0.662	0.197	0.519	0.265	0.281	0.750	0.712
pH - Soil arthro PC1	0.751	0.556	0.829	0.777	0.751	**0.022**	0.777
Plants PC1 - Soil arthro PC2	0.413	0.336	0.137	0.206	0.250	0.894	0.160
Plants PC2 - Soil arthro PC2	0.601	0.907	0.422	0.233	0.497	0.464	0.674
Soil C - Soil arthro PC2	0.590	0.154	**0.014**	0.111	0.300	0.650	0.137
pH - Soil arthro PC2	0.675	0.102	0.675	0.578	0.476	**0.039**	1.000
Soil C - Plants PC1	0.328	**0.025**	0.829	0.260	0.108	0.803	0.200
pH - Plants PC1	0.960	0.829	**0.022**	0.365	0.117	0.138	0.229
Soil C - Plants PC2	0.663	0.364	*0.093*	0.920	0.894	0.532	0.663
pH - Plants PC2	0.777	0.174	0.580	0.627	0.405	0.214	0.276

Supplementary Table 6. Effect of the roof construction and invertebrates on nine ecosystem parameters. Additional exclosures were placed outside the exclosure network at six locations for assessing the potential effects of the "None" exclosure on micro-climatic conditions (called "Micro-climate control" plots). These exclosures were constructed from the same material as the "None" exclosures, but with a 20-cm wide strip of the material used for the "I" plots along the bottom of the eastern side (away from the prevailing weather front), allowing invertebrates to enter while still excluding anything larger. Consequently, the construction assured a comparable microclimate to the "None" exclosure but allowed invertebrates to forage. For details on exclosure construction see main text. Values represent mean (standard error) of data collected, while superscript letters represent statistically different values based on repeated measures ANOVA within a mixed effects modelling framework followed by Tukey's HSD (α = 0.05). "I": Invertebrates have access, "None": No animals have access (see also Fig 1).

Plot name	I	Micro-climate control	None		
Roofed	no	yes	yes		
Herbivores present	invertebrates	invertebrates	none	F	p
Aboveground biomass (g m^{-2})	337.75 (30.54)^{b}	442.12 (49.99)^{b}	602.24 (50.05)^{a}	12.46	<0.001
Maximum vegetation height (cm)	32.95 (1.95)^{b}	31.53 (2.77)^{b}	47.39 (2.15)^{a}	19.06	<0.001
Mean vegetation height (cm)	9.08 (0.62)^{b}	7.24 (0.58)^{b}	15.21 (0.90)^{a}	33.28	<0.001
Soil moisture (%)	34.30 (1.46)^{b}	31.36 (1.37)^{b}	36.42 (1.30)^{a}	4.64	0.010
Air humidity (%)	70.49 (0.72)^{a}	71.27 (0.82)^{a}	72.29 (0.49)^{a}	2.26	0.123
Air temperature (˚C)	11.08 (0.25)^{a}	10.94 (0.29)^{a}	10.85 (0.17)^{a}	0.27	0.762
Soil temperature (˚C)	13.17 (0.27)^{a}	12.77 (0.27)^{ab}	12.36 (0.25)^{b}	3.54	0.030
PAR (µmol m^{-2} s^{-1})	903.16 (72.24)^{a}	712.10 (57.21)^{ab}	611.70 (51.42)^{b}	6.62	0.002
UV (µmol m^{-2} s^{-1})	56.71 (4.13)^{a}	42.04 (3.03)^{b}	37.99 (3.10)^{b}	8.49	<0.001
